# Incidence of cognitive impairment post first stroke: a systematic review and meta-analysis

**DOI:** 10.3389/fneur.2026.1782749

**Published:** 2026-04-13

**Authors:** Xinyu Zhang, Huaqiang Li, Yanyang Li, Xiangwen Hao, Ailipinai Yasen, Xiaoyan Zheng, Yuxin Bai, Jingqi Chen, Qiuhong Man

**Affiliations:** 1 School of Health Preservation and Rehabilitation, Chengdu University of Traditional Chinese Medicine, Chengdu, China; 2 Department of Clinical Laboratory, Shanghai Fourth People’s Hospital, School of Medicine, Tongji University, Shanghai, China; 3 Rehabilitation Medicine Center, The First Affiliated Hospital of Nanjing Medical University, Nanjing, Jiangsu, China

**Keywords:** cognitive impairment, incidence, post-stroke cognitive impairment, stroke, systematic evaluation

## Abstract

**Objective:**

To clarify the incidence of post-stroke cognitive impairment (PSCI) in first-time stroke patients through meta-analysis.

**Methods:**

We conducted a comprehensive search of PubMed, Embase, Web of Science, and the Cochrane Library for studies reporting PSCI incidence up to May 5, 2024. Pooled incidence was calculated using Stata 18.0 with a random-effects model.

**Results:**

A total of 11 publications from six countries with a sample population of 7,048 were included in the final analysis. The overall incidence of PSCI in first-time stroke patients was estimated to be 46% [95% CI (36–57), *I*^2^ = 98.63%]. Subgroup analysis showed that the prevalence of PSCI was 38% in male and 45% in female; The prevalence of PSCI was 54% for hemorrhagic stroke and 44% for ischemic stroke; The prevalence of PSCI was 38% in stroke patients possessing hypertension and 32% in stroke patients without hypertension; The prevalence of PSCI was 44% in stroke patients with diabetes and 36% in stroke patients without diabetes; The prevalence of PSCI was 46% in stroke patients with hyperlipidemia and 40% in stroke patients without hyperlipidemia; The incidence of PSCI in stroke patients aged > 60 years was 44%.

**Conclusion:**

Nearly half of patients with first-time stroke develop PSCI and the rate is higher in females, patients aged > 60 years, hemorrhagic stroke subtype, and those with hypertension, diabetes, and hyperlipidemia compared to males, ischemic stroke subtype, and those without underlying diseases.

**Systematic review registration:**

The protocol for this review was registered with the PROSPERO International Prospective Register of Systematic Reviews (CRD42024611457).

## 1 Introduction

Stroke is the second leading cause of death globally, and in recent years the incidence of stroke has been on the rise among young and middle-aged people, while the risk of stroke gradually increases with age (1). Considering the future trend of population growth and population aging, it is expected that the mortality rate and the number of people with disabilities in stroke patients will rise significantly globally, and the global cost of stroke will be more than $721 billion (0.66% of global GDP) according to recent World Health Stroke Organization statistics (1, 2).

Post-stroke cognitive impairment (PSCI) is any severe cognitive impairment that occurs after a significant stroke, regardless of the cause, and is mainly characterized by a reduction in higher-level functions such as memory, attention, visuospatial, executive, and verbal abilities, which severely affects the patient’s activities of daily living, with incidence rates of up to 60% reported in the literature (3, 4). Importantly, PSCI exists on a severity continuum, with post-stroke dementia (PSD) representing it’s most severe form. Compared to non-PSCI patients, those with PSCI have a significantly higher risk of recurrent stroke (by 59%) and a twofold increase in mortality, imposing a substantial burden on families and the healthcare system (5–7).

It should be noted, however, that the time point of PSCI assessment has previously been highly controversial, with some studies assessing PSCI at 1 week and 2–3 months after stroke, and others assessing PSCI at 3 months, 6 months, and 1 year after stroke (4). It is now internationally accepted that diagnosis of cognitive function should be made 3–6 months after stroke because delirium, which can appear within a week of stroke and continue for up to 3 months. The patient’s condition stabilizes and delirium disappears 3 months after stroke onset allowing for cognitive function to be assessed at this time (3, 4). In addition, Post-stroke dementia (PSD) is different from PSCI, which lies on the severity continuum of clinical manifestations of PSCI (4) and possesses stricter diagnostic criteria and lower incidence. A large number of stroke survivors currently have cognitive impairment, but not enough to fulfill the diagnostic criteria for dementia, but still affects quality of life. Some previous studies of PSCI have assessed PSCI over less than 3 months and have generalized PSD to PSCI, which may lead to bias in the incidence of PSCI. Therefore, our study excluded studies in which the outcome indicator was PSD and the time of assessment was less than 3 months. In terms of cognitive assessment, many previous studies have used different assessment modalities (8), all of which can lead to differences in the studies.

To address these inconsistencies, the American Heart Association/American Stroke Association (AHA/ASA) published a Scientific Statement recommending standardized tools for PSCI assessment after ischemic and hemorrhagic stroke (3). This statement identifies the Mini-Mental State Examination (MMSE) and Montreal Cognitive Assessment (MoCA) as the most widely validated and recommended screening instruments. In our meta-analysis, we adopted these guidelines to ensure diagnostic consistency and reduce heterogeneity arising from variable assessment methods.

In summary, our study aims to explore the incidence of PSCI in patients with first-time stroke through meta-analysis, and provide evidence for early prevention and treatment of PSCI.

## 2 Method

### 2.1 Registration and enrollment

This systematic review and meta-analysis was conducted in 2024 and followed the guidelines of the Preferred Reporting Items for Systematic Reviews and Meta-Analyses (PRISMA) (9). The protocol for this review was registered with the PROSPERO International Prospective Register of Systematic Reviews (CRD42024611457).

### 2.2 Inclusion and exclusion criteria

The inclusion and exclusion criteria for this systematic review are summarized in Table 1.

**Table 1:** Inclusion and exclusion criteria.

Category	Criteria
Inclusion criteria	1. The study was conducted in patients with a first stroke
	2. Cognitive function assessed at 3 months post-stroke (studies reporting data at 3 months were prioritized; studies only reporting later time points were excluded).
	3. Pre-stroke cognitive impairment was excluded
	4. PSCI diagnosed using assessment tools recommended in the AHA/ASA Scientific Statement (3), specifically the Mini-Mental State Examination (MMSE) or Montreal Cognitive Assessment (MoCA)
	5. Both cohort and cross-sectional studies were eligible for inclusion.
	6. No language restriction
Exclusion criteria	1. Inability to determine whether the study population was a first stroke
	2. Studies in which the outcome indicator was post-stroke dementia
	3. Unable to extract raw data
	4. Sample size < 50 participants

We excluded studies focusing on post-stroke dementia (PSD) because PSD represents the severe end of the PSCI spectrum, diagnosed using stricter criteria (e.g., DSM or ICD) and involving different clinical trajectories, notably impairment in daily activities. Including PSD-specific studies could underestimate the broader burden of early cognitive deficits and introduce clinical heterogeneity. Studies with fewer than 50 participants were excluded to ensure statistical stability and precision of effect estimates.

### 2.3 Literature search strategy

We conducted a comprehensive literature search across four electronic databases, including PubMed, Embase, Web of Science (WOS), and The Cochrane Library, to identify eligible studies on post-stroke cognitive impairment (PSCI) in first-ever stroke patients. The search was performed with no language restrictions, and the time scope covered all publications from the establishment of each database to May 5, 2024.

The core retrieval strategy was constructed based on two key subject clusters: stroke and cognitive dysfunction, with the Boolean logic operator AND used for the combination of the two clusters, and OR for the expansion of synonyms and variant expressions within each cluster. The core search terms for stroke included “Stroke,” “Cerebral Stroke,” “Acute Stroke” and their plural forms; the core search terms for cognitive dysfunction included “Cognitive Dysfunction,” “Cognitive Disorder,” “Dementia” and their plural forms. Mesh terms (for PubMed) and field-limited retrieval (e.g., title/abstract for Embase, topic search for WOS) were applied in corresponding databases to improve the accuracy and comprehensiveness of retrieval.

The detailed, step-by-step search formulas for each database, including the specific combination of subject headings, free words and field restrictions, are presented in Appendix 1 for full transparency and reproducibility of the study.

### 2.4 Literature screening and data extraction

Screening was done independently by two authors (XZ, HL). The full text of these studies was then obtained and independently assessed by two investigators (XZ, HL) to assess their eligibility for inclusion in the systematic evaluation. Any disagreements were resolved through consensus discussions with senior expert (QM). For studies that used more than one guideline-recommended assessment tool, we further verified whether the study provided a clear definition of cognitive impairment (e.g., specified cut-off values). If such a definition was lacking, the study was excluded after consultation with a third expert (QM). This step was necessary because different tools have varying diagnostic thresholds, and unclear case definitions could introduce heterogeneity. Full texts in non-English languages were translated using DeepL Translator (DeepL GmbH, Cologne, Germany) to ensure accurate comprehension of study eligibility and data extraction. Data were extracted independently by 2 authors (XZ, HL), including first author, year of publication, country, total sample size, patient age, diagnostic tools, and counts of cases with PSCI. For studies reporting cognitive assessment data at multiple time points (e.g., 6 months, 9 months, 12 months), we prioritized data from the time point closest to 3 months post-stroke for analysis. Studies that only provided data at time points beyond 4 months (e.g., 6, 9, or 12 months) and did not report 3-month data were excluded to ensure temporal consistency of the outcome measure, consistent with our inclusion criterion 2.

### 2.5 Quality assessment

Independent quality assessment of the included studies was performed by two authors (HL, HT). Cross-sectional studies were evaluated using the Agency for Healthcare Research and Quality (AHRQ) methodology checklist (10). This tool assesses study quality across 11 domains, including sample representativeness, data collection methods, and control for confounding factors. Each domain is scored as 1 for “yes” (met the criterion) or 0 for “no” or “unclear” (did not meet or insufficient information). Total scores range from 0 to 11, with scores of 8–11 indicating high quality, 4–7 indicating moderate quality, and 0–3 indicating low quality.

Cohort and case–control studies were evaluated for quality using the Newcastle-Ottawa Scale (NOS) (10), which consists of 8 entries with a total score of 9 points, mainly including the selection of the study population (0–4 points), between-group comparability (0–2 points), and the measurement of the exposure factors (0–3 points), and a score of 7–9 points is considered to be of high quality, 5–6 points is considered to be of moderate quality, and 0–4 points are considered to be of low quality.

### 2.6 Statistical analysis

Data synthesis and statistical analyses were conducted using Stata version 18.0 (StataCorp LP, College Station, TX, USA). We calculated the pooled prevalence of PSCI with its corresponding 95% confidence interval (CI). Heterogeneity across studies was assessed using the Cochran’s Q test, and the I^2^ statistic was calculated to quantify the proportion of total variation due to heterogeneity. Heterogeneity was considered small when *p* > 0.1 and *I*^2^ < 50%, in which case a fixed-effects model was used for pooling. Heterogeneity was considered large when *p* ≤ 0.1 and *I*^2^ ≥ 50%, and a random-effects model was applied. To explore potential sources of heterogeneity, we conducted sensitivity analyses and predefined subgroup analyses. Potential publication bias was assessed by visual inspection of a funnel plot for asymmetry and further corroborated by both Begg’s rank correlation test and Egger’s linear regression test. *p* < 0.05 was considered a statistically significant difference.

### 2.7 Meta-regression analysis

To further investigate potential sources of heterogeneity, we performed a univariate meta-regression analysis using Stata 18.0 to assess the extent to which prespecified moderating variables explained variability in PSCI incidence. Moderating variables were selected based on the literature and data availability, and were categorized as follows:

(1) Categorical variables: cognitive assessment tool (MMSE, MoCA, or both), stroke type (ischemic, hemorrhagic, or mixed), geographic region (Asia, Europe, Africa), and study design (cohort or cross-sectional);
(2) Continuous variables: sample size, mean age, and proportions of patients with hypertension and diabetes.

Stepwise regression was used to identify statistically significant moderators, with entry and removal probabilities set at *p* < 0.1 and *p* > 0.1, respectively. The contribution of each variable to heterogeneity was evaluated using regression coefficients (*β*), 95% confidence intervals, and corresponding *p* values.

## 3 Results

### 3.1 Results of the literature search

**Figure 1:**
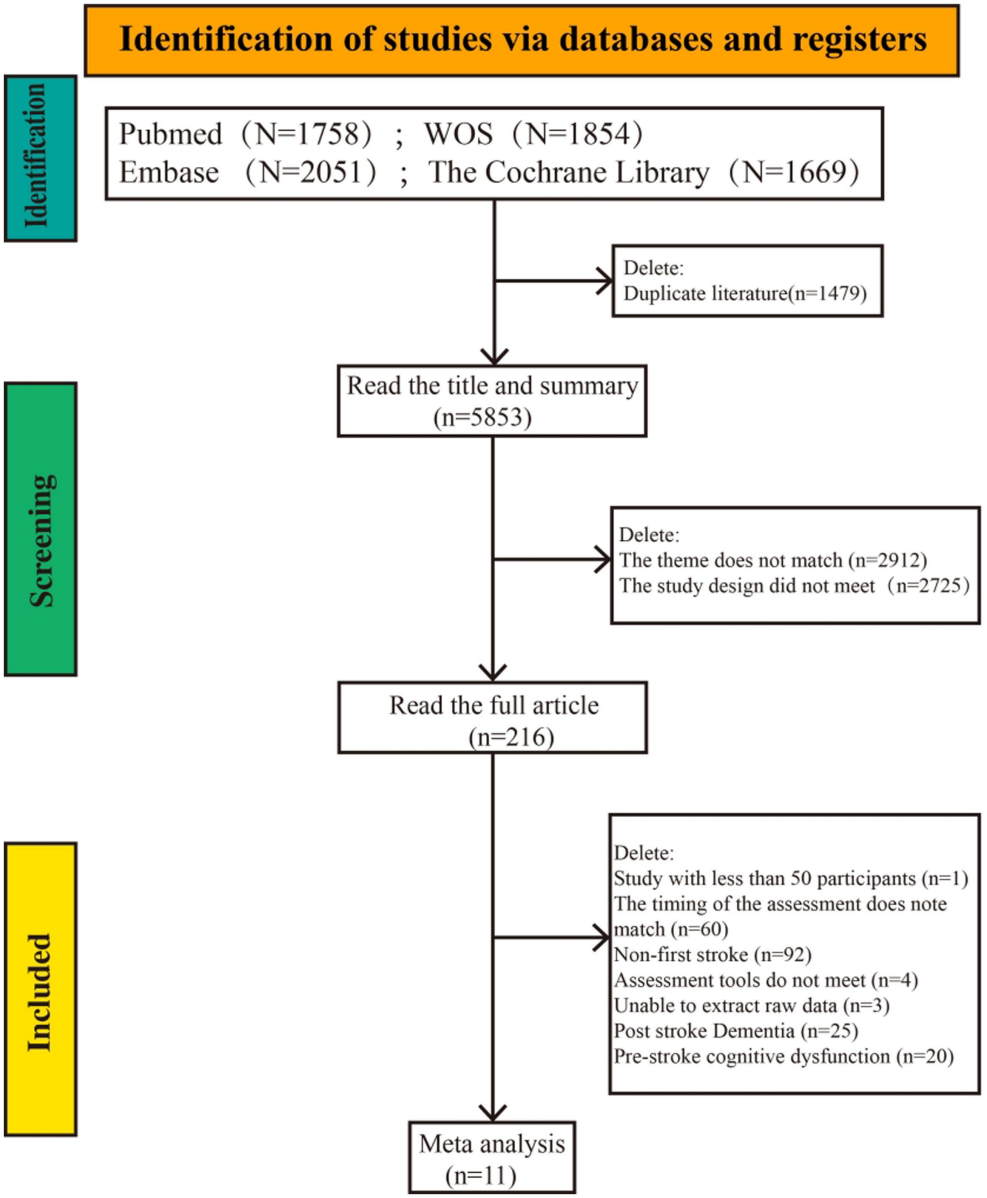
Literature screening flowchart. ES, effect size; CI, confidence interval.

The systematic search of PubMed, Embase, Web of Science, and the Cochrane Library yielded a total of 7,332 records. After removing 1,479 duplicates, 5,853 records remained for title and abstract screening, of which 5,637 were excluded. The remaining 216 full-text articles were assessed for eligibility, and 205 were excluded for reasons including: not reporting first stroke data, outcome measure not PSCI, assessment time point outside 3 months, or sample size < 50. Ultimately, 11 studies from six countries, comprising 7,048 participants, were included in the final meta-analysis (11–21). The literature screening process is presented in the PRISMA flow diagram (Figure 1). The characteristics of the included studies are summarized in Table 2.

**Table 2:** Characteristics of included studies.

Author	Country	Stroke type	Year	Total Age (years)	Age PSCI (years)	Age No-PSCI (years)	Sample	PSCI n (%)	Study design	Assessment tools	Quality evaluation
Liman et al. (19)	Ireland	Ischemic	2011^b^		74.4 ± 9.9	69.5 ± 13.1	630	93	Cohort	MMSE	8
Wong et al. (15)	China	Mixed	2012^b^	54 ± 11^a^			90	66	Cohort	MoCA	7
Tu et al. (14)	China	Ischemic	2014^b^	≥40^a^			689	288	Cross-sectional	MoCA/MMSE	8
Jacquin et al. (20)	France	Ischemic	2014^b^		73.5 ± 13.4	59.4 ± 16.0	220	104	Cohort	MMSE/MoCA	7
Yoon et al. (21)	Korea	Ischemic	2017^b^	≥19^a^			2,625	890	Cohort	MMSE	7
He et al. (13)	China	Ischemic	2018^b^	40–75^a^			708	509	Cohort	MoCA	7
Jia et al. (16)	China	Ischemic	2020^b^		65.6 ± 11.1	62.3 ± 10.5	1,019	523	Cohort	MMSE	8
Esmael et al. (17)	Egypt	Mixed	2021^b^		68.5 ± 7.12	60.23 ± 7.61	150	38	Cohort	MoCA	7
Xu et al. (12)	China	Mixed	2023^b^		67.75 ± 12.66	67.13 ± 11.45	311	120	Cohort	MoCA	7
Huang et al. (11)	China	Ischemic	2023^b^	≥60^a^			483	195	Cross-sectional	MMSE	7
Boutros et al. (18)	Lebanon	Hemorrhagic	2023^b^	73.7 ± 12^a^			123	92	Cohort	MMSE	8

a Age profile of the total population included in the study; MMSE: Mini-Mental State Examination; MoCA: Montreal Cognitive Assessment; PSCI: Post-stroke cognitive impairment; No-PSCI: No post-stroke cognitive impairment.

b Studies are ordered chronologically by year of publication. For studies that did not report age separately for PSCI and non-PSCI groups, only total population age is shown.

### 3.2 Basic characteristics and quality assessment of the included literature

A total of 11 studies comprising 7,048 participants were included. Among these, seven studies enrolled only patients with ischemic stroke, one study enrolled only patients with hemorrhagic stroke, and three studies included both ischemic and hemorrhagic stroke patients. Eleven studies were found to be of moderate to high quality after rigorous literature quality evaluation. Detailed characteristics of the included studies are presented in Table 2, and the complete quality assessment results for each study are provided in Appendix 2.

### 3.3 Meta-analysis results

The pooled incidence of post-stroke cognitive impairment (PSCI) following a first-ever stroke was 46% (95% CI 36%–57%), with substantial heterogeneity observed across the 7,048 individuals from the included studies (*I*^2^ = 98.63%, *p* < 0.01).

**Figure 2:**
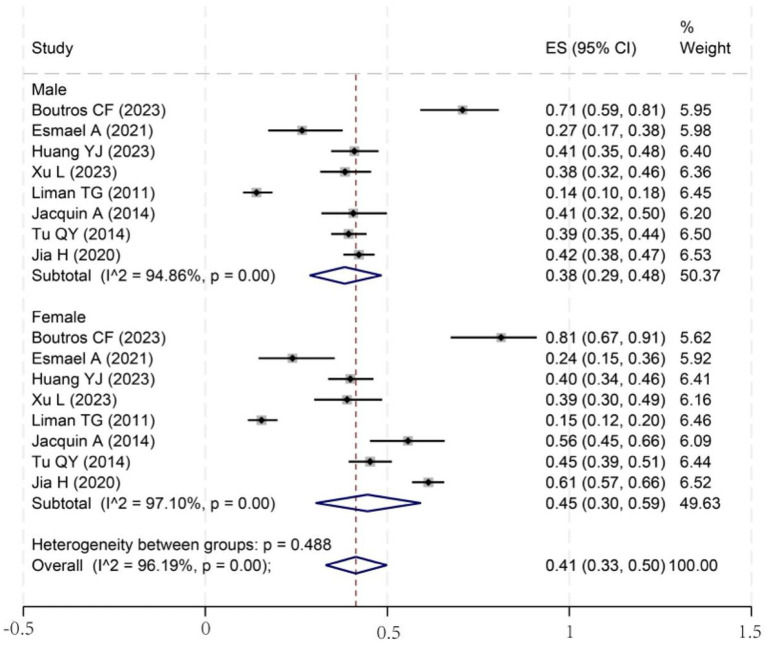
Forest plot for subgroup analysis of sex differences. ES, effect size; CI, confidence interval.

**Figure 3:**
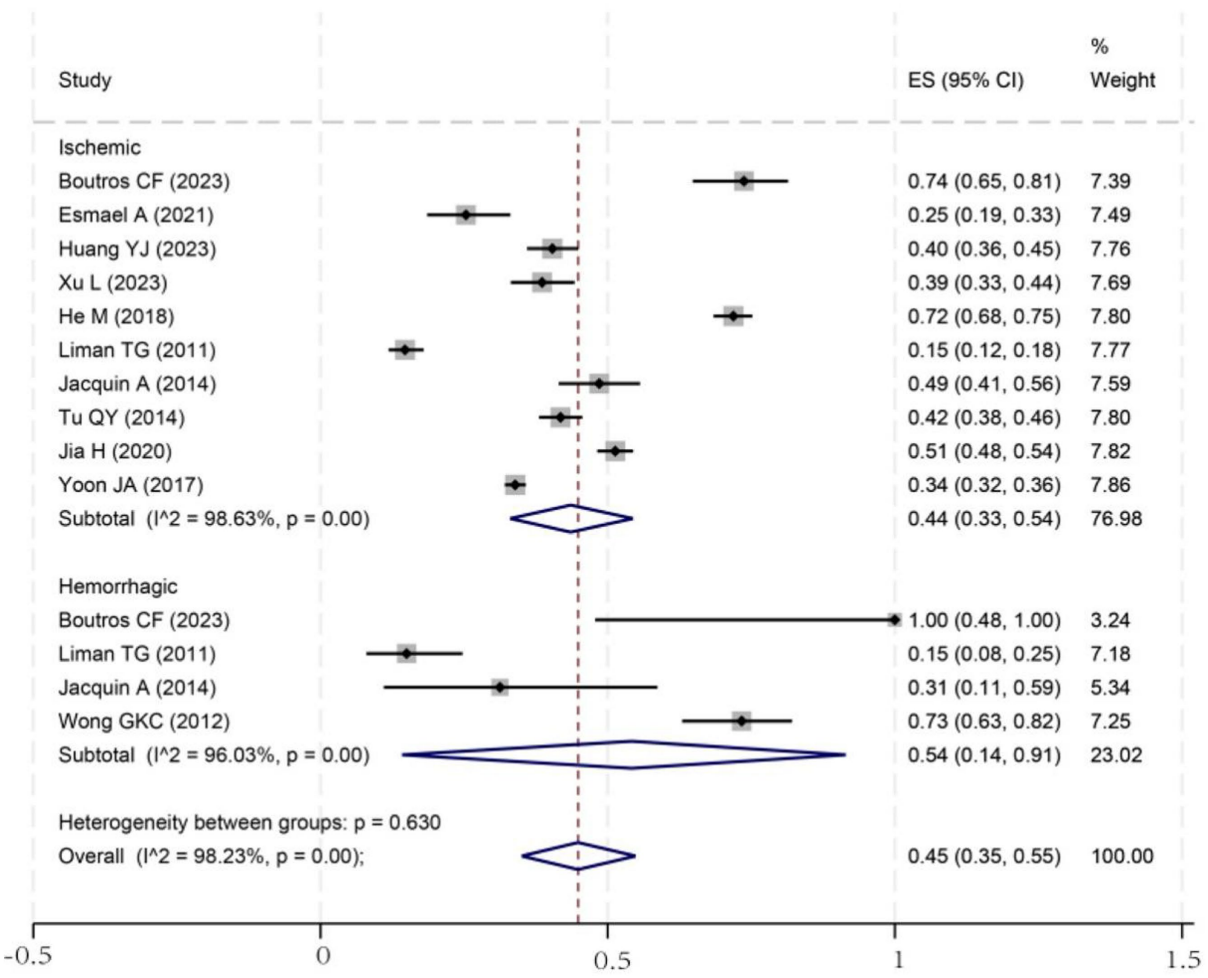
Forest plot of stroke type subgroup analysis. ES, effect size; CI, confidence interval.

**Figure 4:**
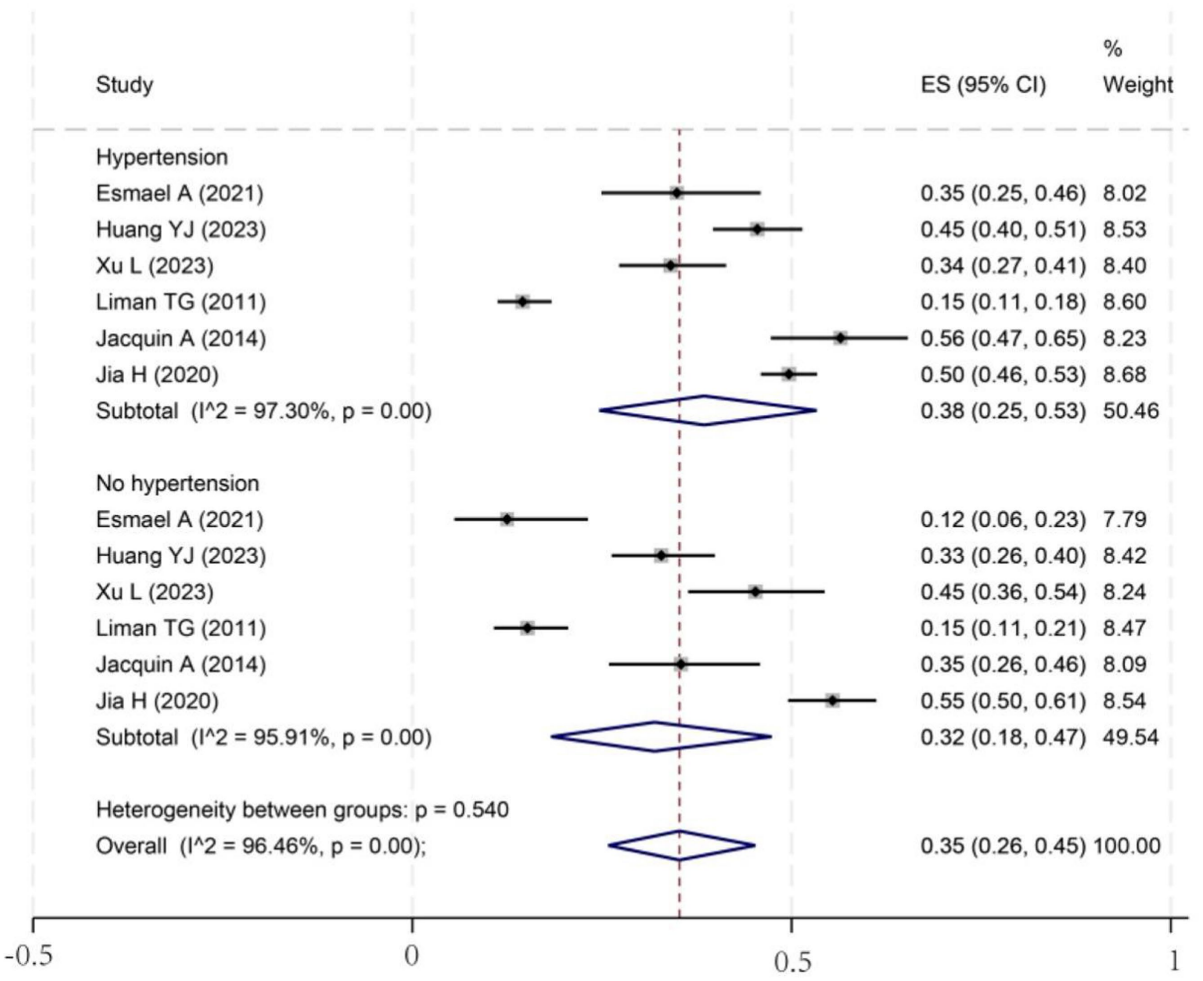
Forest plot for subgroup analysis of hypertension. ES, effect size; CI, confidence interval.

**Figure 5:**
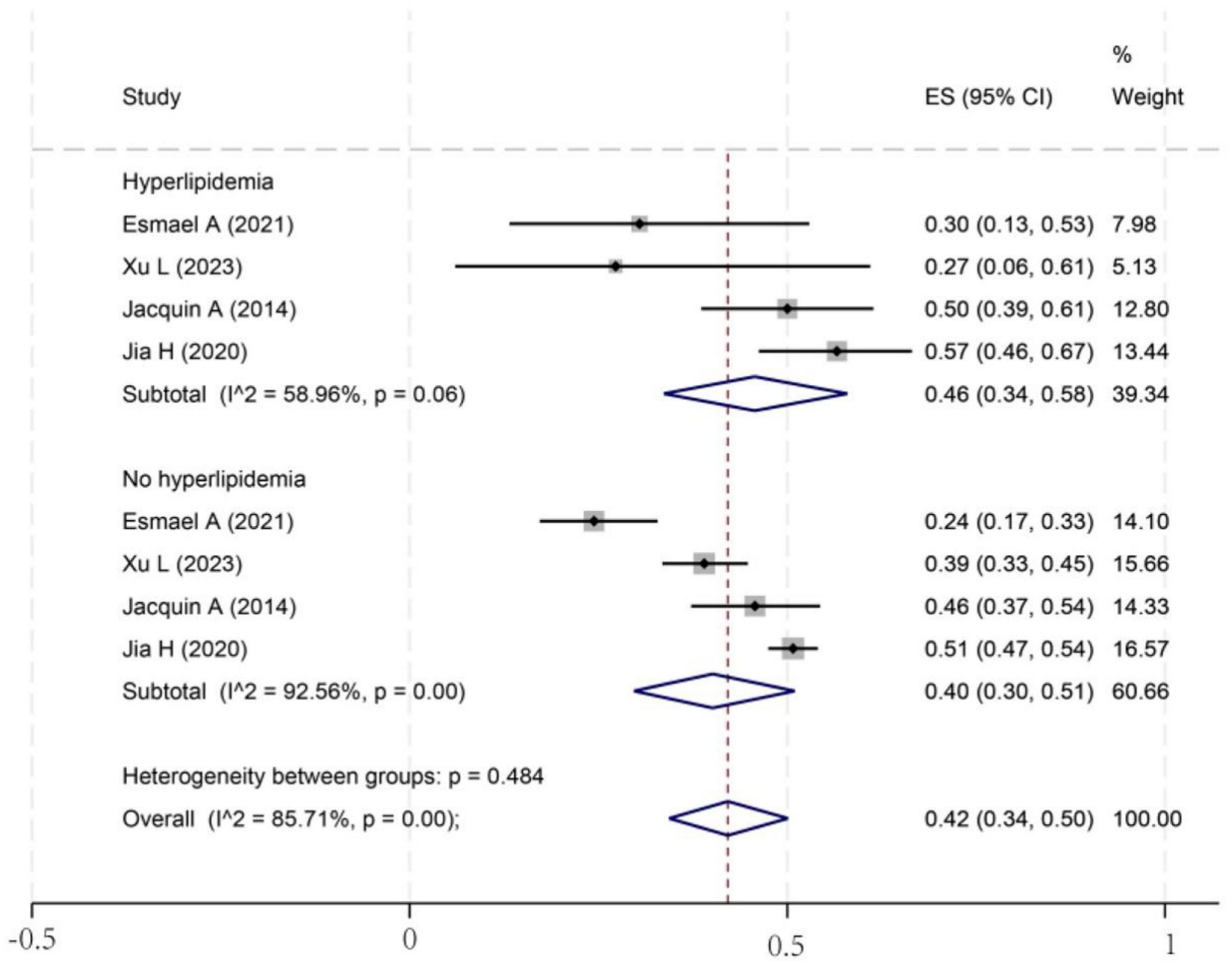
Forest plot for hyperlipidemia subgroup analysis. ESES, effect size; CI, confidence interval.

**Figure 6:**
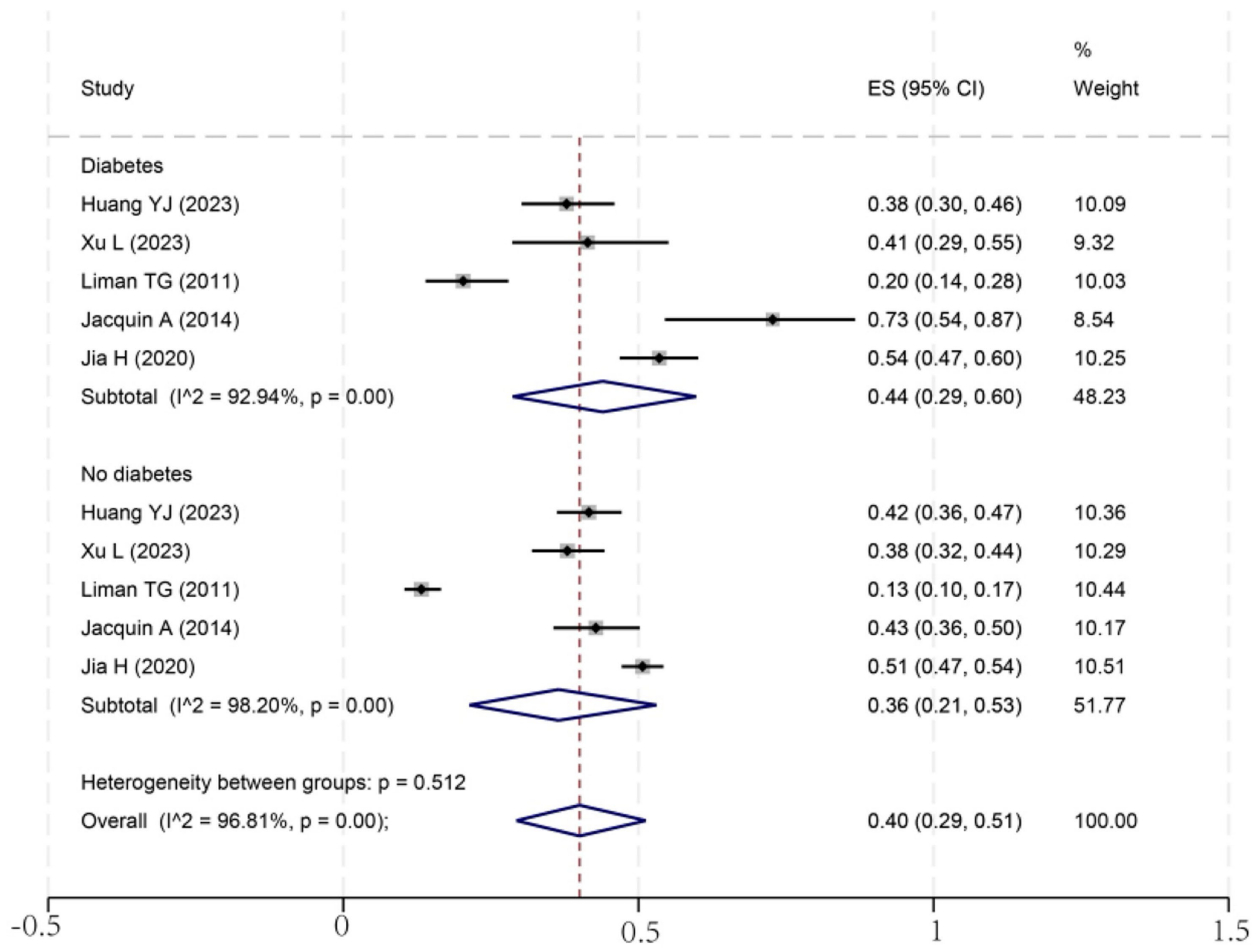
Forest plot for diabetes subgroup analysis. ES, effect size; CI, confidence interval.

**Figure 7:**
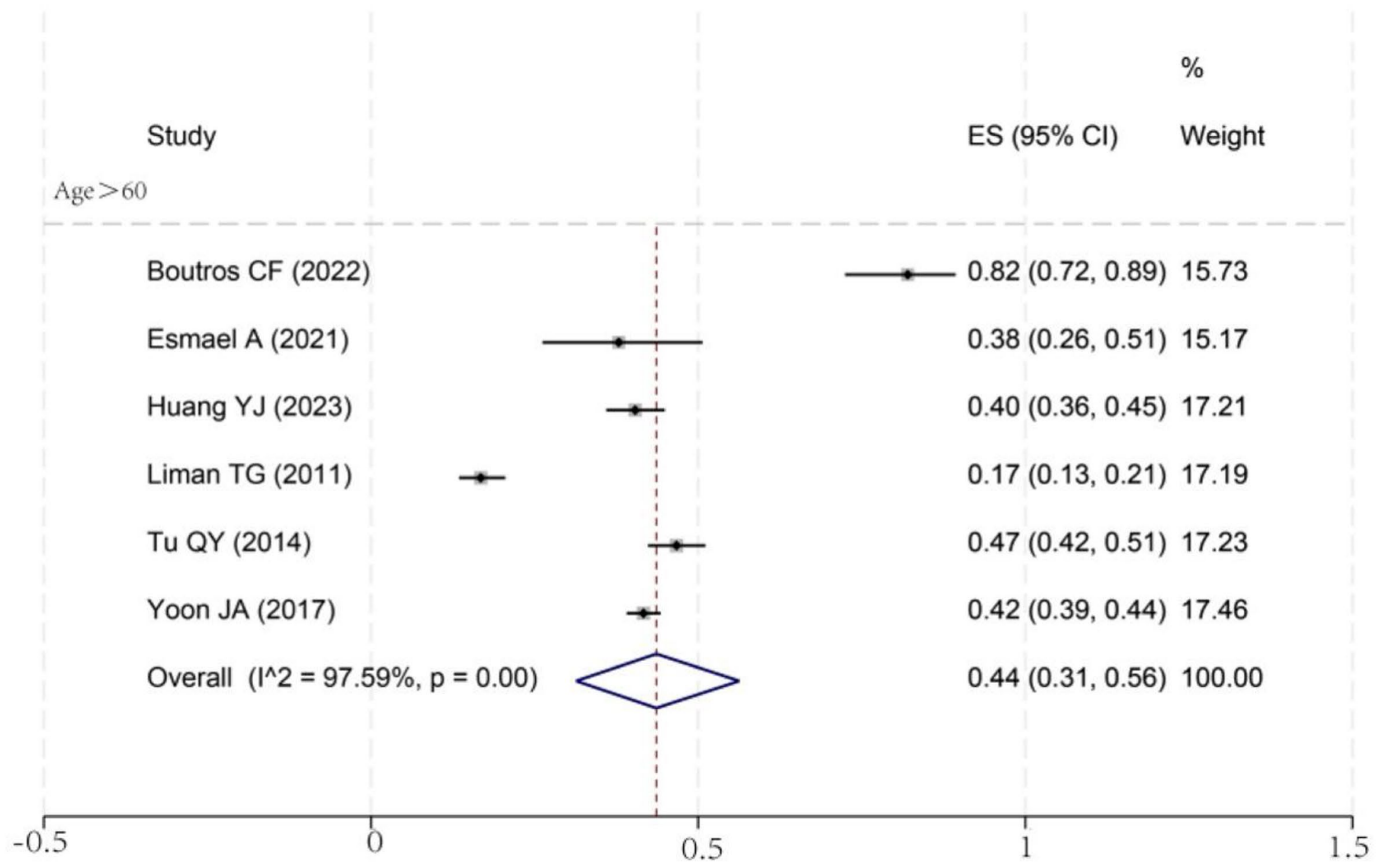
Forest map for age subgroup analysis. ES, effect size; CI, confidence interval.

**Figure 8:**
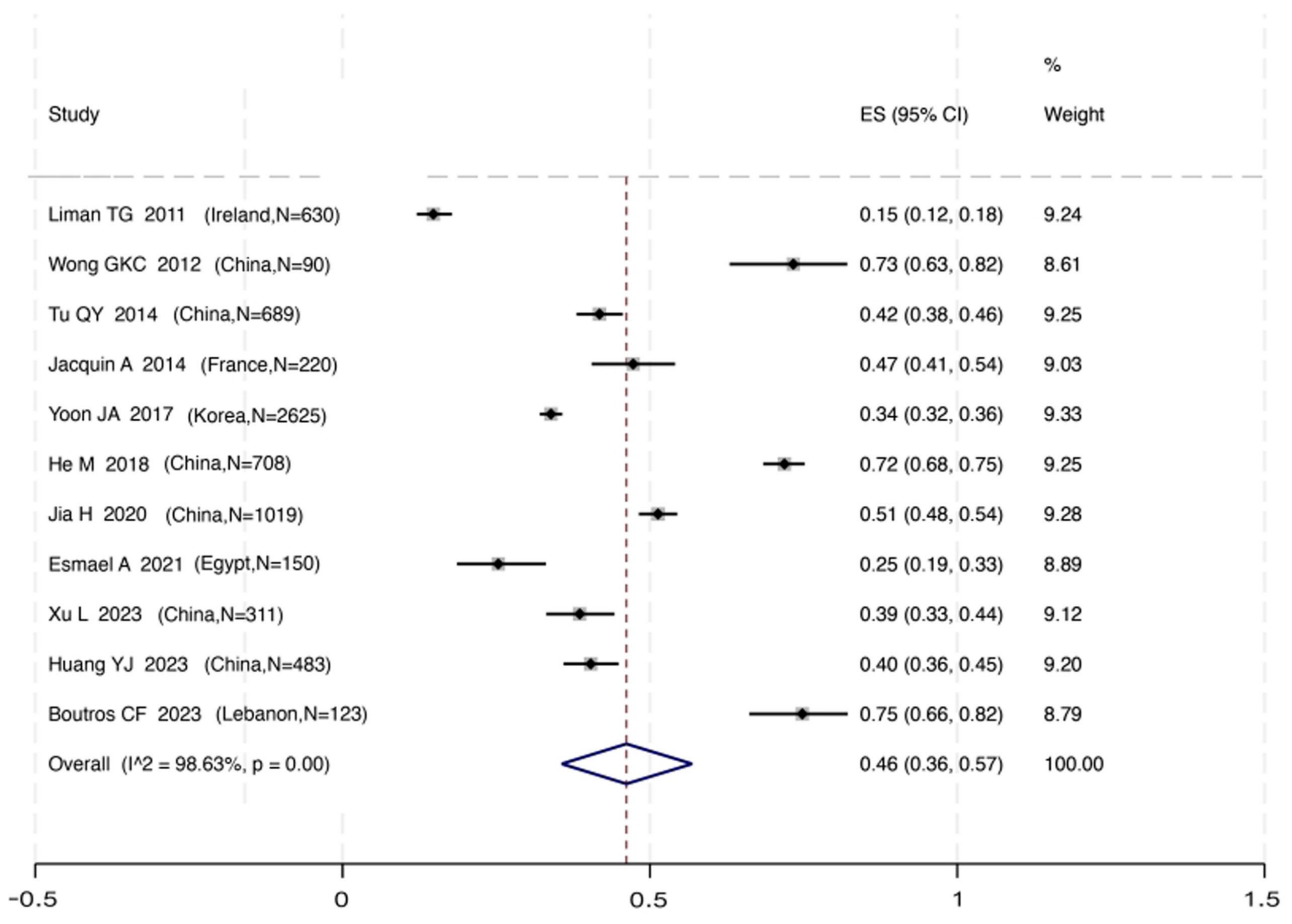
Forest plot of incidence of cognitive impairment after the first stroke. CI, confidence interval.

To investigate potential sources of this heterogeneity, we performed a series of subgroup analyses using data from studies that reported relevant stratifications. The results are summarized below and presented in Figures 2–7. By sex (Figure 2), the incidence was 38% in males [95% CI (29–48), *I*^2^ = 94.86%, *p* < 0.01] and 45% in females [95% CI (30–59), *I*^2^ = 97.1%, *p* < 0.01]. By stroke type (Figure 3), the incidence was 54% for hemorrhagic stroke [95% CI (14–91), *I*^2^ = 96.03%, *p* < 0.01] and 44% for ischemic stroke [95% CI (33–54), *I*^2^ = 98.63%, *p* < 0.01]. Regarding comorbidities (Figures 4–6), patients with hypertension had a higher incidence [38, 95% CI (25–53), *I*^2^ = 97.3%, *p* < 0.01] than those without [32, 95% CI (18–47), *I*^2^ = 95.91%, *p* < 0.01]. Similarly, patients with diabetes showed a higher incidence [44, 95% CI (29–60), *I*^2^ = 92.94%, *p* < 0.01] than those without [36, 95% CI (21–53), *I*^2^ = 98.2%, *p* < 0.01]. For hyperlipidemia, the incidence was 46% in affected patients [95% CI (34–58), *I*^2^ = 58.96%, *p* > 0.05]. and 40% in those without [95% CI (30–51), *I*^2^ = 92.56%, *p* < 0.01]. By age (Figure 7), patients over 60 years had an incidence of 44% [95% CI (31–56), *I*^2^ = 97.59%, *p* < 0.01]. The forest plot of the incidence of cognitive impairment after the first stroke is shown in Figure 8. Although subgroup analyses by sex, stroke type, comorbidities, etc. did not eliminate high heterogeneity (Table 3), they provided a basis for subsequent meta-regression analyses to screen potential moderating variables. This study further identified cognitive assessment tools through meta-regression, study regions as key sources of heterogeneity affecting PSCI incidence.

**Table 3:** Results of subgroup analysis.

Classification	Number of studies included	Sample size	Heterogeneous results		Results	95% CI
			I*^2^*	*p*		
Sex						
Male (11, 12, 14, 16–20)	8	1,948	94.86	< 0.01	0.38	0.29–48
Female (11, 12, 14, 16–20)	8	1,677	97.1	< 0.01	0.45	0.3–0.59
Types of strokes						
Ischemic stroke (11–14, 16–21)	10	6,857	98.63	< 0.01	0.44	0.33–0.54
Hemorrhagic stroke (15, 18–20)	4	191	96.03	< 0.01	0.54	0.14–0.91
Hypertension						
Yes (11, 12, 16, 17, 19, 20)	6	1,812	97.3	< 0.01	0.38	0.25–0.53
No (11, 12, 16, 17, 19, 20)	6	1,001	95.91	< 0.01	0.32	0.18–0.47
Diabetes						
Yes (11, 12, 16, 19, 20)	5	613	92.94	< 0.01	0.44	0.29–0.60
No (11, 12, 16, 19, 20)	5	2,050	98.2	< 0.01	0.36	0.21–0.53
Hyperlipidemia						
Yes (12, 16, 17, 20)	4	213	58.96	> 0.05	0.46	0.34–0.58
No (12, 16, 17, 20)	4	1,487	92.56	< 0.01	0.4	0.3–0.51
Age>60 (9, 13, 16–18, 20)	6	3,044	97.59	< 0.01	0.44	0.31–0.56

All pooled estimates were calculated using a random-effects model due to substantial heterogeneity (*I*^2^ > 50%). CI, confidence interval.

#### 3.3.1 Sensitivity analysis

**Figure 9:**
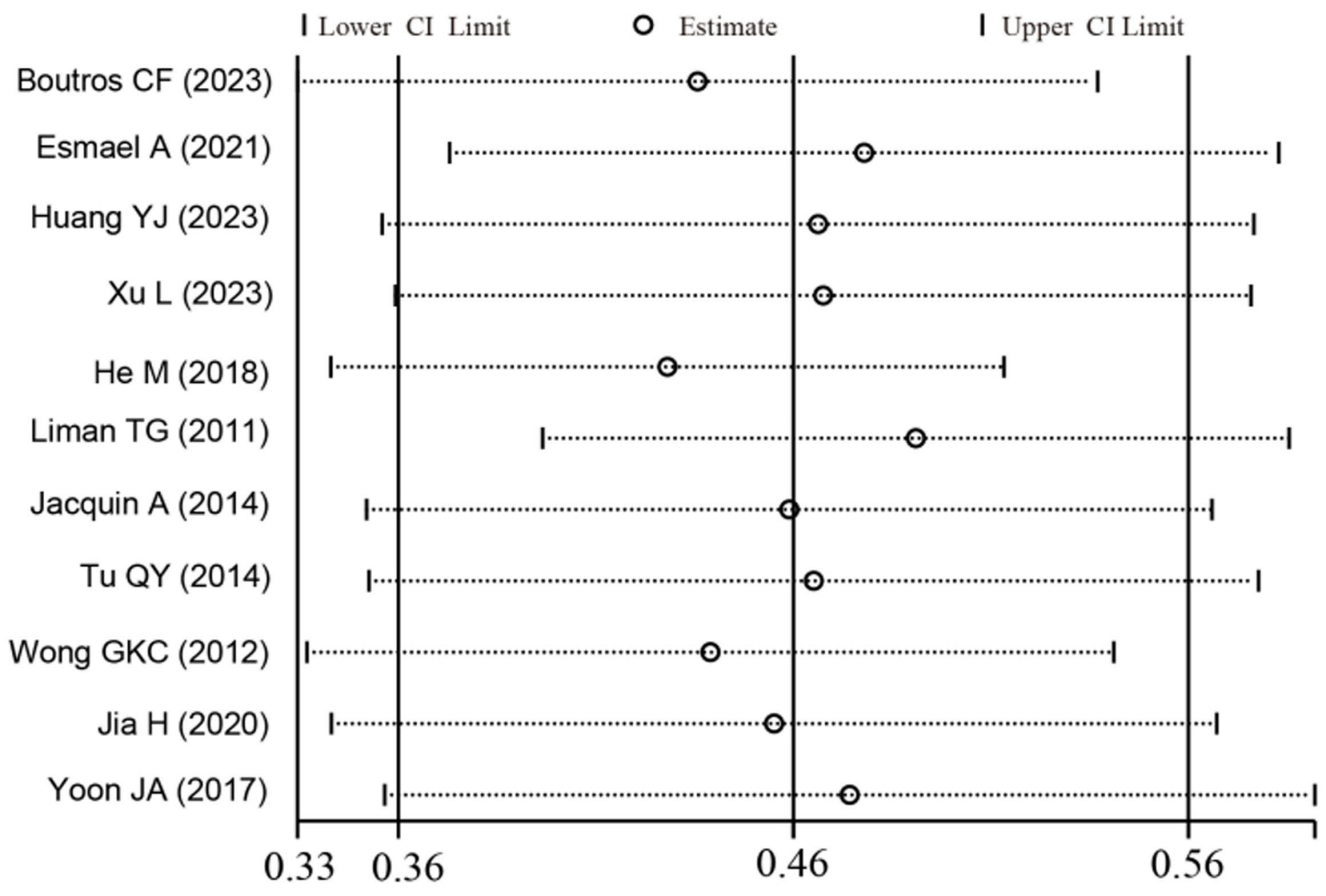
Sensitivity analysis of first-time stroke patients. ES, effect size.

Sensitivity analysis demonstrated that excluding any single study did not significantly alter the pooled effect size, indicating that the meta-analysis results were robust and not disproportionately influenced by any individual study. The results of the sensitivity analysis are shown in Figure 9.

#### 3.3.2 Publication bias test

**Figure 10:**
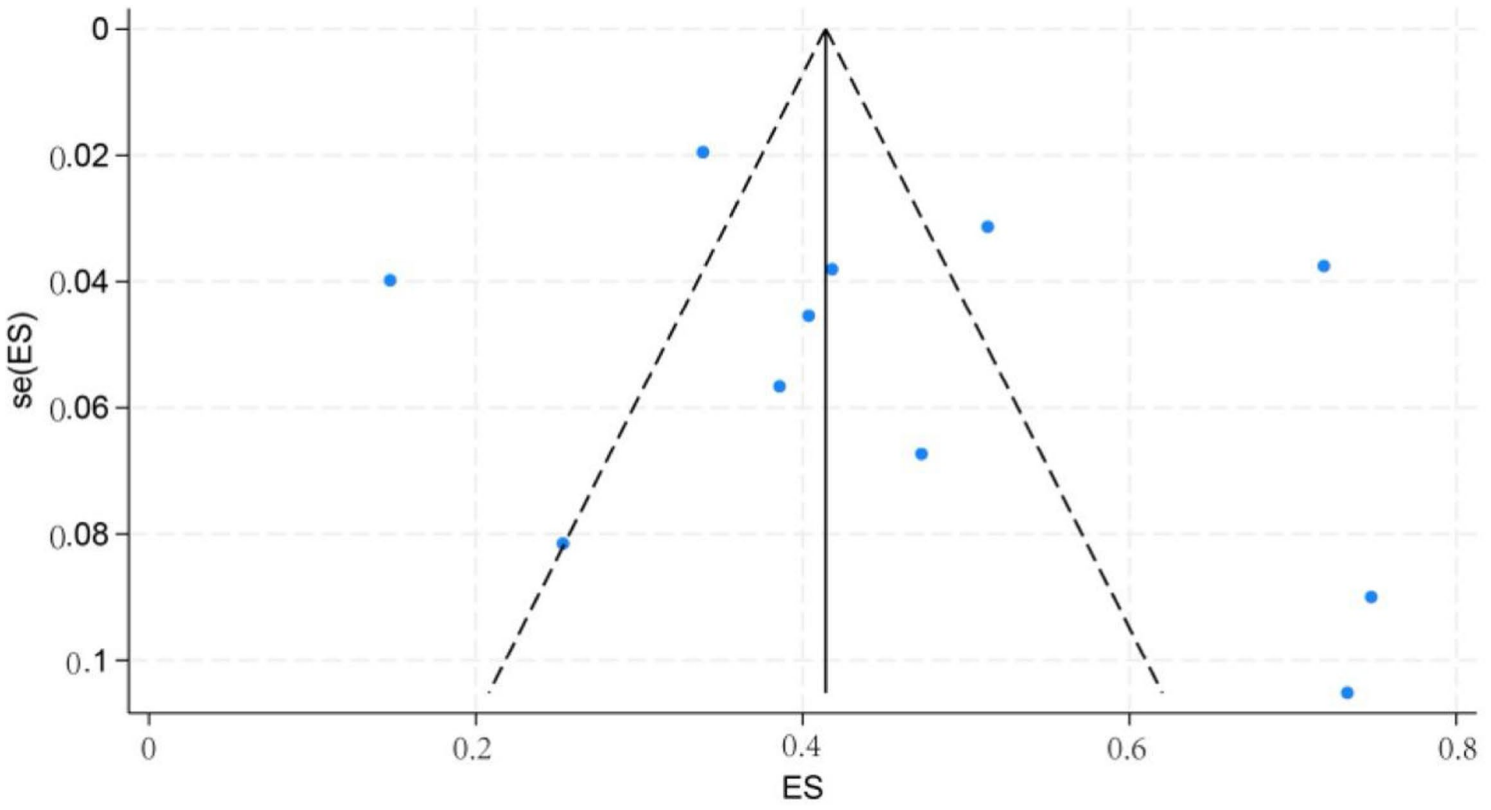
Funnel plot of incidence of cognitive impairment in patients with first stroke.

Publication bias was assessed using Begg’s and Egger’s tests. For all subgroup analyses, both Begg’s test (*p* > 0.05) and Egger’s test (*p* > 0.05) indicated no significant publication bias, supporting the stability of the pooled estimates. The funnel plot is shown in Figure 10.

### 3.4 Results of meta-regression analysis

To clarify the source of high heterogeneity in the pooled analysis of the incidence of PSCI after the first stroke in this study (*I*^2^ = 98.63%, *p* < 0.01), univariate and multivariate stepwise meta-regression analyses were conducted using Stata 18.0, including eight potential moderating variables. The results are as follows.

#### 3.4.1 Single-factor meta-regression results

Univariate analysis showed that cognitive assessment tools and study areas were significant sources of heterogeneity in this study (all *p* < 0.05), and the average age of patients was a potential source of heterogeneity (*p* = 0.089). The other variables had no significant explanatory effect on heterogeneity (*p* all > 0.1).

#### 3.4.2 Results of multivariate stepwise meta-regression

Eight moderating variables were included in the multiple stepwise regression model (inclusion criterion: *p* < 0.1; exclusion criterion: *p* > 0.1). Ultimately, only cognitive assessment tools (*β* = 0.125, *p* = 0.009) and study location (*β* = 0.098, *p* = 0.021) were retained in the final model. The model’s coefficient of determination, *R*^2^ = 0.286, indicates that these two variables combined can explain 28.6% of the inter-study heterogeneity in this study; the overall model test *p* = 0.008, demonstrating the statistical reliability of the variables in explaining heterogeneity.

Further examination of the variable interaction showed that there was no significant interaction between cognitive assessment tools and the study area (*p* = 0.312), indicating that the two effects on the incidence of PSCI are independent of each other, and there is no synergistic effect of “a specific assessment tool in a certain area has a higher detection rate.” The remaining 71.4% of heterogeneity was unexplained, presumably related to raw data limitations of the included literature (Table 4).

**Table 4:** Results of meta-regression analysis of PSCI incidence after first stroke.

Moderator variable	Type	Regression coefficient (β)	95% CI	*p*-value	Explains heterogeneity
Cognitive assessment tool	Categorical	0.125	0.032 ~ 0.218	0.009	Yes
Study region	Categorical	0.098	0.015 ~ 0.181	0.021	Yes
Mean patient age	Continuous	0.006	0.000 ~ 0.012	0.089	Potential
Sample size	Continuous	0.000	−0.001 ~ 0.001	0.756	No
Stroke type	Categorical	0.052	−0.041 ~ 0.145	0.273	No
Study design	Categorical	0.036	−0.058 ~ 0.130	0.451	No
Proportion with hypertension	Continuous	0.002	−0.003 ~ 0.007	0.412	No
Proportion with diabetes	Continuous	0.003	−0.004 ~ 0.010	0.405	No

Cognitive assessment tool (cognitive assessment tool) assignment: 1 = MMSE, 2 = MoCA, 3 = MMSE+MoCA; Study region (study region) assignment: 1 = Asia, 2 = Europe, 3 = Africa; Stroke type (stroke type) assignment: 1 = ischemic, 2 = hemorrhagic, 3 = mixed; Study design (study design) assignment: 1 = cohort study, 2 = cross-sectional study; β is the regression coefficient, and the positive value indicates that the higher the level of this variable, the higher the incidence of PSCI after the first stroke.

## 4 Discussion

In this systematic review and meta-analysis, we found that the incidence of PSCI in first-time stroke patients was 46% (95% CI 36%–57%). This figure is lower than the approximately 60% often cited in clinical guidelines (3). We propose several key factors may explain this discrepancy. First, our analysis was strictly limited to patients with a first-ever stroke, a criterion not always emphasized in the foundational studies that informed the guidelines. Given that a history of stroke is a significant risk factor for recurrent cognitive events (22), the inclusion of patients with recurrent strokes would likely inflate the overall incidence. Second, a critical methodological distinction lies in the difference between incidence and prevalence. Some studies in the literature did not exclude pre-stroke cognitive impairment, meaning that they reported prevalence rather than incidence (3). This conflation inevitably elevates the reported figures, likely accounting for the discrepancy between guideline values and the lower incidence derived from our strictly defined cohort. Finally, the guideline’s figure may be derived from data with limited geographical scope, whereas our analysis incorporated data from six countries, reflecting a broader spectrum of healthcare contexts and population characteristics. Our data exhibited significant regional variations, exemplified by the low 3-month incidence of only 14.8% reported in the Irish cohort (19). This suggests that differences in healthcare systems, diagnostic criteria, and population risk factors across different countries can significantly influence the overall pooled estimates. The substantial heterogeneity observed in our pooled analysis (*I*^2^ = 98.63%) warrants careful consideration. Rather than viewing this solely as a statistical limitation, it should be recognized as reflecting genuine clinical and methodological diversity across the included studies. This variability likely arises from differences in baseline population risk (e.g., vascular risk factors), stroke characteristics (e.g., subtype, lesion location and size), and post-stroke management protocols (e.g., acute treatment, rehabilitation) across different settings. Our meta-regression and subgroup analyses were designed to explore these sources of variation and confirmed that factors such as cognitive assessment tool and geographic region contributed significantly to the observed heterogeneity. Contextualizing this heterogeneity is essential for interpreting our pooled estimate: the 46% incidence figure represents an average across diverse populations and methods, not a fixed estimate applicable to all clinical settings.

A review of the baseline characteristics revealed that nearly half (43.2%) of the included population was over 60 years of age. Consequently, we conducted a subgroup analysis to examine the relationship between age and PSCI onset. We found that the incidence of PSCI in individuals older than 60 was 44%, which is comparable to the overall pooled incidence. This suggests that age remains a critical determinant of post-stroke cognitive outcomes. However, this finding warrants cautious interpretation. Given that cognitive decline is often a physiological consequence of aging, it is difficult to ascertain whether the impairment observed in older adults is solely attributable to the stroke event itself or represents a combination of stroke and age-related decline. This diagnostic ambiguity is further complicated by the lack of universally accepted, age-adjusted cut-off values for commonly used screening tools like the MMSE and MoCA in clinical practice. Previous studies indicate that the use of previously fixed cut-off values to assess cognitive function in older adults is inappropriate and that cut-off value adjustments should be made (23, 24). More in-depth studies are needed to establish more robust methods for assessing baseline cognitive function in the elderly population.

We found that the incidence of PSCI was significantly lower in men than in women, consistent with most previous studies (25, 26). However, discrepancies remain; for instance, a study by Sarah T et al. reported no significant sex difference in PSCI rates (27). Importantly, that study noted that the sensitivity of the Brief Mental Status Examination was higher in women while specificity was lower (27), suggesting that the diagnostic accuracy of screening tools may vary by sex. Additionally, the specific cognitive domains assessed by a test can significantly influence the detection of PSCI in men versus women. The MMSE primarily focuses on verbal abilities, whereas the MoCA places greater emphasis on executive function and visuoconstructive tasks, covering a broader range of cognitive domains. Whereas men tend to perform better on naming tests after the onset of injury, women tend to perform better on verbal memory and category fluency tests (28). Consequently, the differential use of MMSE and MoCA across the studies included in our meta-analysis may have contributed to the observed sex differences. However, it is important to acknowledge that we were unable to perform a separate subgroup analysis based solely on the assessment tool due to the limited number of studies available. Therefore, the potential confounding effect arising from the distinct psychometric properties of the MMSE and MoCA remains a limitation of our study. In addition, Bako et al. suggested that variations in luteinizing hormone, estradiol, and neurotrophic factors between sexs could modulate cognitive recovery post-stroke (29). Despite these insights, the relationship between sex and PSCI remains debated, necessitating further studies to clarify these mechanisms. We observed numerically higher incidence of PSCI was observed in patients with hemorrhagic stroke than in patients with ischemic stroke, which is consistent with that reported in the AHA/ASA guideline (3). In addition, a systematic evaluation by Syed et al. reported a 55% prevalence of PSCI within 6 months after cerebral hemorrhage (30); notably, our observed incidence of 54% at 3 months aligns closely with these figures. Cerebral hemorrhage is an acute cerebrovascular disease compared to cerebral ischemia, in which blood overflows and forms hematomas in the brain due to rupture of blood vessels in the brain, a condition that often leads to rapid deterioration of the condition and direct compression and damage to the surrounding brain tissues and neurons (31), thereby precipitating cognitive impairment. Furthermore, secondary injury mechanisms play a crucial role. Studies have demonstrated that iron deposition in critical brain regions, such as the hippocampus, increases significantly following cerebral hemorrhage (32). This accumulation elevates reactive oxygen species (ROS) levels, which down-regulates Itga3 gene expression and accelerates the depletion of the hippocampal neural stem cell pool, ultimately leading to cognitive impairment (33, 34). These pathophysiological mechanisms provide a plausible explanation for the higher PSCI incidence observed in hemorrhagic stroke patients in our analysis.

Our subgroup analysis revealed that the incidence of PSCI was higher in patients with hypertension (38%) compared to those without (32%). This finding aligns with the well-established understanding - stroke patients are often accompanied by symptoms of hypertension (35). The long-term effects of the blood pressure load of hypertension on the small blood vessels of the brain can lead to pathological changes such as vitrification and lumen narrowing of the small blood vessels (36). These changes cause an increase in cerebral circulatory resistance and insufficient blood perfusion to important memory function areas of the brain (37). This leads to chronic hypoxia and inadequate nutrient supply to the brain that precipitate cognitive impairment. In addition, the whole brain volume and hippocampal volume of hypertensive patients are reduced compared with those with normal blood pressure (38), and the hippocampus is an important region of the brain responsible for memory and cognitive functions, and its reduced volume will directly affect the cognitive function of the patients. Hypertension can also reduce hippocampal connectivity, leading to cognitive deficits (39). When stroke occurs in hypertensive patients, the brain tissue damage caused by stroke is often more serious because the cerebrovascular lesions already exist. Consequently, the brain’s resilience is compromised, and the capacity for cognitive recovery post-stroke may be significantly limited.

It is well known that hyperlipidemia is one of the important risk factors for atherosclerosis (40). Hyperlipidemic patients have excessive lipid content in the blood, which is easily deposited on the blood vessel wall, forming atherosclerotic plaques (41). These plaques will gradually increase in size, leading to the narrowing of the blood vessel lumen, affecting the blood supply to the brain, and the long-term low perfusion state will damage the function and structure of brain cells, increasing the risk of cognitive impairment after stroke (42). Similarly, diabetes mellitus is a primary cause of microangiopathy, which can impair cerebral microvasculature, leading to tissue ischemia and subsequent cognitive dysfunction. Critically, these conditions often coexist; diabetic patients frequently present with metabolic abnormalities, including hyperlipidemia and hypertension, which synergistically accelerate atherosclerosis and exacerbate cerebral blood flow insufficiency (42). Interestingly, our meta-analysis provides a unique statistical perspective on this pathophysiological link. We observed that the subgroup of patients with hyperlipidemia exhibited considerably lower heterogeneity (I^2^ = 58.96%, *p* > 0.05) compared to all other subgroups, which consistently showed extreme heterogeneity (I^2^ > 90%). This finding suggests that the impact of hyperlipidemia on PSCI may be driven by a more consistent underlying mechanism. It is plausible that the pro-atherosclerotic and ischemic effects of hyperlipidemia represent a dominant pathway for cognitive decline after stroke, thus reducing the influence of other variables that contribute to inter-study variability.

Our study has several strengths. 1. The epidemiological data provided by systematic review and meta-analysis are the most up-to-date data on the incidence of PSCI in patients with a first stroke. 2. Due to the use of stringent inclusion and exclusion criteria, the reports that we have included from the available literature provide an accurate estimate of the incidence of PSCI, which is most representative of the characteristics of the incidence of PSCI in the different regions after a first stroke.

This study has several limitations: 1. Ideally, valid estimates of the combined incidence of PSCI would require inverse probability weighting using population weights, which was not done in this meta-analysis. 2. Although our meta-regression identified cognitive assessment tool and geographic region as significant contributors to heterogeneity, they explained only 28.6% of the variance, leaving 71.4% unexplained. This residual heterogeneity likely reflects unmeasured differences in factors such as stroke severity, education level, lesion characteristics, and post-stroke care protocols. The persistence of substantial heterogeneity highlights the multifactorial nature of PSCI and suggests that our pooled estimate should be interpreted as an average across diverse populations, not a precise prediction for any specific group. 3. Sample sizes for some subgroup analyses are too small and may affect the accuracy of the results. 4. Our inclusion criteria were limited to studies using the MMSE or MoCA for cognitive assessment. While this approach improved diagnostic consistency and reduced heterogeneity, it may have excluded large-scale studies that used other validated screening tools. As a result, our pooled prevalence estimates might not fully reflect findings from those alternative instruments. Future research should include a broader range of validated tools to assess whether the choice of screening instrument affects prevalence estimates.

## 5 Conclusion

This meta-analysis demonstrates that nearly half of first-ever stroke survivors develop PSCI within 3 months, underscoring the substantial burden of cognitive impairment in this population. Incidence is notably higher among females, older adults (>60 years), patients with hemorrhagic stroke, and those with vascular risk factors including hypertension, diabetes, and hyperlipidemia. These findings highlight the importance of routine cognitive screening in high-risk subgroups and support the development of targeted early intervention strategies to mitigate long-term disability and improve quality of life in stroke survivors.

## Data Availability

The raw data supporting the conclusions of this article will be made available by the authors, without undue reservation.

## Supplementary material

APPENDIX 1 Literature search strategies.

APPENDIX 2 Literature quality assessment.

## References

[1] Feigin VL Owolabi MO. Pragmatic solutions to reduce the global burden of stroke: a world stroke organization-lancet neurology commission. Lancet Neurol. (2023) 22:1160–206. doi: 10.1016/S1474-4422(23)00277-6, 37827183 PMC10715732

[2] Feigin VL Brainin M Norrving B Martins S Sacco RL Hacke W . World stroke organization (WSO): global stroke fact sheet 2022. Int J Stroke. (2022) 17:18–29. doi: 10.1177/17474930211065917, 34986727

[3] El Husseini N Katzan IL Rost NS Blake ML Byun E Pendlebury ST . Cognitive impairment after ischemic and hemorrhagic stroke: a scientific statement from the American Heart Association/American Stroke Association. Stroke. (2023) 54:e272–91. doi: 10.1161/STR.0000000000000430, 37125534 PMC12723706

[4] Rost NS Brodtmann A Pase MP van Veluw SJ Biffi A Duering M . Post-stroke cognitive impairment and dementia. Circ Res. (2022) 130:1252–71. doi: 10.1161/circresaha.122.319951, 35420911

[5] Oksala NK Jokinen H Melkas S Oksala A Pohjasvaara T Hietanen M . Cognitive impairment predicts poststroke death in long-term follow-up. J Neurol Neurosurg Psychiatry. (2009) 80:1230–5. doi: 10.1136/jnnp.2009.174573 19620138

[6] Rohde D Gaynor E Large M Mellon L Hall P Brewer L . The impact of cognitive impairment on Poststroke outcomes: a 5-year follow-up. J Geriatr Psychiatry Neurol. (2019) 32:275–81. doi: 10.1177/0891988719853044, 31167593

[7] Dowling NM Johnson S Nadareishvili Z. Poststroke cognitive impairment and the risk of recurrent stroke and mortality: systematic review and meta-analysis. J Am Heart Assoc. (2024) 13:e033807. doi: 10.1161/JAHA.123.033807, 39239841 PMC11935622

[8] Stolwyk RJ O'Neill MH McKay AJ Wong DK. Are cognitive screening tools sensitive and specific enough for use after stroke? A systematic literature review. Stroke. (2014) 45:3129–34. doi: 10.1161/STROKEAHA.114.004232 25074518

[9] Page MJ McKenzie JE Bossuyt PM Boutron I Hoffmann TC Mulrow CD . The PRISMA 2020 statement: an updated guideline for reporting systematic reviews. BMJ. (2021) 372:n71. doi: 10.1136/bmj.n71, 33782057 PMC8005924

[10] Meyer GS Battles J Hart JC Tang N. The US agency for healthcare research and quality's activities in patient safety research. Int J Qual Health Care. (2003) 15:i25–30. doi: 10.1093/intqhc/mzg068 14660520

[11] Huang Y Wang Q Zou P He G Zeng Y Yang J. Prevalence and factors influencing cognitive impairment among the older adult stroke survivors: a cross-sectional study. Front Public Health. (2023) 11:1254126. doi: 10.3389/fpubh.2023.1254126, 37790718 PMC10542404

[12] Xu L Xiong Q Du Y Huang LW Yu M. Nonlinear relationship between glycated hemoglobin and cognitive impairment after acute mild ischemic stroke. BMC Neurol. (2023) 23:116. doi: 10.1186/s12883-023-03158-x, 36949414 PMC10031995

[13] He M Wang J Liu N Xiao X Geng S Meng P . Effects of blood pressure in the early phase of ischemic stroke and stroke subtype on poststroke cognitive impairment. Stroke. (2018) 49:1610–7. doi: 10.1161/strokeaha.118.020827, 29895539

[14] Tu Q Ding B Yang X Bai S Tu J Liu X . The current situation on vascular cognitive impairment after ischemic stroke in Changsha. Arch Gerontol Geriatr. (2014) 58:236–47. doi: 10.1016/j.archger.2013.09.006, 24148887

[15] Wong GK Lam S Ngai K Wong A Mok V Poon WS. Evaluation of cognitive impairment by the Montreal cognitive assessment in patients with aneurysmal subarachnoid haemorrhage: prevalence, risk factors and correlations with 3 month outcomes. J Neurol Neurosurg Psychiatry. (2012) 83:1112–7. doi: 10.1136/jnnp-2012-302217, 22851612

[16] Jia H Li H Liu Y Liu C Xue M. Elevated serum alkaline phosphatase as a predictor of cognitive impairment in patients with acute ischaemic stroke: a retrospective cohort study. Arch Gerontol Geriatr. (2020) 89:104104. doi: 10.1016/j.archger.2020.104104, 32460124

[17] Esmael A Elsherief M Eltoukhy K. Prevalence of cognitive impairment in acute ischaemic stroke and use of Alberta stroke Programme early CT score (ASPECTS) for early prediction of post-stroke cognitive impairment. Neurol Neurochir Pol. (2021) 55:179–85. doi: 10.5603/PJNNS.a2021.0006, 33507530

[18] Boutros CF Khazaal W Taliani M Sadier NS Salameh P Hosseini H. Factors associated with cognitive impairment at 3, 6, and 12 months after the first stroke among Lebanese survivors. Brain Behav. (2023) 13:e2837. doi: 10.1002/brb3.2837, 36495111 PMC9847618

[19] Liman TG Heuschmann PU Endres M Flöel A Schwab S Kolominsky-Rabas PL. Changes in cognitive function over 3 years after first-ever stroke and predictors of cognitive impairment and long-term cognitive stability: the Erlangen stroke project. Dement Geriatr Cogn Disord. (2011) 31:291–9. doi: 10.1159/000327358, 21502760

[20] Jacquin A Binquet C Rouaud O Graule-Petot A Daubail B Osseby GV . Post-stroke cognitive impairment: high prevalence and determining factors in a cohort of mild stroke. J Alzheimer's Dis. (2014) 40:1029–38. doi: 10.3233/JAD-131580, 24577459

[21] Yoon JA Kim DY Sohn MK Lee J Lee SG Lee YS . Factors associated with improvement or decline in cognitive function after an ischemic stroke in Korea: the Korean stroke cohort for functioning and rehabilitation (KOSCO) study. BMC Neurol. (2017) 17:9. doi: 10.1186/s12883-016-0780-3, 28073355 PMC5223558

[22] Filler J Georgakis MK Dichgans M. Risk factors for cognitive impairment and dementia after stroke: a systematic review and meta-analysis. Lancet Healthy Longev. (2024) 5:e31–44. doi: 10.1016/S2666-7568(23)00217-9, 38101426

[23] Zhang S Qiu Q Qian S Lin X Yan F Sun L . Determining appropriate screening tools and cutoffs for cognitive impairment in the Chinese elderly. Front Psych. (2021) 12:773281. doi: 10.3389/fpsyt.2021.773281, 34925100 PMC8674928

[24] Elkana O Tal N Oren N Soffer S Ash EL. Is the cutoff of the MoCA too high? Longitudinal data from highly educated older adults. J Geriatr Psychiatry Neurol. (2020) 33:155–60. doi: 10.1177/0891988719874121, 31500493

[25] Mellon L Brewer L Hall P Horgan F Williams D Hickey A. Cognitive impairment six months after ischaemic stroke: a profile from the ASPIRE-S study. BMC Neurol. (2015) 15:31. doi: 10.1186/s12883-015-0288-2, 25879880 PMC4359388

[26] Dong L Briceno E Morgenstern LB Lisabeth LD. Poststroke cognitive outcomes: sex differences and contributing factors. J Am Heart Assoc. (2020) 9:e016683. doi: 10.1161/JAHA.120.016683, 32633589 PMC7660722

[27] Exalto LG Weaver NA Kuijf HJ Aben HP Bae HJ Best JG . Sex differences in Poststroke cognitive impairment: a multicenter study in 2343 patients with acute ischemic stroke. Stroke. (2023) 54:2296–303. doi: 10.1161/STROKEAHA.123.042507, 37551589 PMC10453354

[28] Brandt E Singh S Bowren M Bhagvathi A Tranel D Boes AD. The role of gender in cognitive outcomes from stroke. J Int Neuropsychol Soc. (2023) 29:878–84. doi: 10.1017/S1355617723000036, 36781414 PMC10757593

[29] Bako AT Potter T Tannous J Pan AP Johnson C Baig E . Sex differences in post-stroke cognitive decline: a population-based longitudinal study of nationally representative data. PLoS One. (2022) 17:e0268249. doi: 10.1371/journal.pone.0268249, 35522611 PMC9075630

[30] Kazim SF Ogulnick JV Robinson MB Eliyas JK Spangler BQ Hough TJ . Cognitive impairment after intracerebral hemorrhage: a systematic review and meta-analysis. World Neurosurg. (2021) 148:141–62. doi: 10.1016/j.wneu.2021.01.026, 33482414

[31] Wan Y Holste KG Hua Y Keep RF Xi G. Brain edema formation and therapy after intracerebral hemorrhage. Neurobiol Dis. (2023) 176:105948. doi: 10.1016/j.nbd.2022.105948, 36481437 PMC10013956

[32] Galea I Durnford A Glazier J Mitchell S Kohli S Foulkes L . Iron deposition in the brain after aneurysmal subarachnoid hemorrhage. Stroke. (2022) 53:1633–42. doi: 10.1161/STROKEAHA.121.036645 35196874

[33] Haller S Bartsch A Nguyen D Rodriguez C Emch J Gold G . Cerebral microhemorrhage and iron deposition in mild cognitive impairment: susceptibility-weighted MR imaging assessment. Radiology. (2010) 257:764–73. doi: 10.1148/radiol.10100612, 20923870

[34] Zhang X Li H Wang H Zhang Q Deng X Zhang S . Iron/ROS/Itga3 mediated accelerated depletion of hippocampal neural stem cell pool contributes to cognitive impairment after hemorrhagic stroke. Redox Biol. (2024) 71:103086. doi: 10.1016/j.redox.2024.103086, 38367510 PMC10883838

[35] Buonacera A Stancanelli B Malatino L. Stroke and hypertension: an appraisal from pathophysiology to clinical practice. Curr Vasc Pharmacol. (2019) 17:72–84. doi: 10.2174/1570161115666171116151051, 29149815

[36] Iadecola C Gottesman RF. Neurovascular and cognitive dysfunction in hypertension. Circ Res. (2019) 124:1025–44. doi: 10.1161/CIRCRESAHA.118.313260, 30920929 PMC6527115

[37] Cipolla MJ Liebeskind DS Chan SL. The importance of comorbidities in ischemic stroke: impact of hypertension on the cerebral circulation. J Cereb Blood Flow Metab. (2018) 38:2129–49. doi: 10.1177/0271678X18800589, 30198826 PMC6282213

[38] Triantafyllou A Ferreira JP Kobayashi M Micard E Xie Y Kearney-Schwartz A . Longer duration of hypertension and MRI microvascular brain alterations are associated with lower hippocampal volumes in older individuals with hypertension. J Alzheimer's Dis. (2020) 74:227–35. doi: 10.3233/JAD-190842, 32039844 PMC7175941

[39] Feng R Rolls ET Cheng W Feng J. Hypertension is associated with reduced hippocampal connectivity and impaired memory. EBioMedicine. (2020) 61:103082. doi: 10.1016/j.ebiom.2020.103082, 33132184 PMC7585137

[40] Suh JS Kim SYJ Lee SH Kim RH Park NH. Hyperlipidemia is necessary for the initiation and progression of atherosclerosis by severe periodontitis in mice. Mol Med Rep. (2022) 26:273. doi: 10.3892/mmr.2022.12789, 35795972 PMC9309540

[41] Gaggini M Gorini F Vassalle C. Lipids in atherosclerosis: pathophysiology and the role of calculated lipid indices in assessing cardiovascular risk in patients with hyperlipidemia. Int J Mol Sci. (2022) 24:75. doi: 10.3390/ijms24010075, 36613514 PMC9820080

[42] Li B Lu X Moeini M Sakadžić S Thorin E Lesage F. Atherosclerosis is associated with a decrease in cerebral microvascular blood flow and tissue oxygenation. PLoS One. (2019) 14:e0221547. doi: 10.1371/journal.pone.0221547, 31469849 PMC6716780

