# Supplementary material for: Incidence of cognitive impairment post first stroke: a systematic review and meta-analysis
Source: Front Neurol. 2026 Apr 13;17:1782749. doi: 10.3389/fneur.2026.1782749 (PMC13111006; doi:10.3389/fneur.2026.1782749)
Supplement: APPENDIX 1 — Literature search strategies. [file Supplementary_file_1.doc]

**Search strategy**

# Pubmed search：

| Step | Search formula |
| --- | --- |
| #1 | "Stroke"[Mesh] |
| #2 | Strokes[Title/Abstract] OR Cerebral Stroke[Title/Abstract] OR Cerebral Strokes[Title/Abstract] OR Stroke, Cerebral [Title/Abstract] OR Strokes, Cerebral[Title/Abstract] OR Stroke, Acute[Title/Abstract] OR Acute Stroke[Title/Abstract] OR Acute Strokes[Title/Abstract] OR Strokes, Acute[Title/Abstract] |
| #3 | #1OR#2 |
| #4 | "Cognitive Dysfunction"[Mesh] |
| #5 | Cognitive Dysfunctions[Title/Abstract] OR Dysfunction, Cognitive[Title/Abstract] OR Dysfunctions, Cognitive[Title/Abstract] OR Cognitive Disorder[Title/Abstract] OR Cognitive Disorders[Title/Abstract] OR Disorder, Cognitive[Title/Abstract] OR Dementia[Title/Abstract] OR Dementias[Title/Abstract] |
| #6 | #4OR#5 |
| #7 | #3AND#6 |

# Embase search：

| Step | Search formula |
| --- | --- |
| #1 | 'Stroke':ab,ti OR 'Strokes':ab,ti OR 'Cerebral Stroke':ab,ti OR 'Cerebral Strokes':ab,ti OR 'Stroke, Cerebral':ab,ti OR 'Strokes, Cerebral':ab,ti OR 'Stroke, Acute':ab,ti OR 'Acute Stroke':ab,ti OR 'Acute Strokes':ab,ti OR 'Strokes, Acute':ab,ti |
| #2 | 'Cognitive Dysfunction':ab,ti OR 'Cognitive Dysfunctions':ab,ti OR 'Dysfunction, Cognitive':ab,ti OR 'Dysfunctions, Cognitive':ab,ti OR 'Cognitive Disorder':ab,ti OR 'Cognitive Disorders':ab,ti OR 'Disorder, Cognitive':ab,ti OR 'Dementia':ab,ti OR 'Dementias':ab,ti |
| #4 | #1 AND #2 |

# Web of science search:

| Step | Search formula |
| --- | --- |
| #1 | TS=(Stroke OR Strokes OR Cerebral Stroke OR Cerebral Strokes OR Stroke, Cerebral OR Strokes, Cerebral OR Stroke, Acute OR Acute Stroke OR Acute Strokes OR Strokes, Acute) |
| #2 | TS=(Cognitive Dysfunction OR Cognitive Dysfunctions OR Dysfunction, Cognitive OR Dysfunctions, Cognitive OR Cognitive Disorder OR Cognitive Disorders OR Disorder, Cognitive OR Dementia OR Dementias ) |
| #3 | #1 AND #2 |

# The Cochrane Library search ：

| Step | Search formula |
| --- | --- |
| #1 | "Stroke" |
| #2 | (Strokes):ab,ti,kw OR (Cerebral Stroke):ab,ti,kw OR (Cerebral Strokes):ab,ti,kw OR (Stroke, Cerebral):ab,ti,kw OR (Strokes, Cerebral):ab,ti,kw OR (Stroke, Acute):ab,ti,kw OR (Acute Stroke):ab,ti,kw OR (Acute Strokes):ab,ti,kw OR (Strokes, Acute):ab,ti,kw |
| #3 | #1OR#2 |
| #4 | "Cognitive Dysfunction" |
| #5 | (Cognitive Dysfunctions):ab,ti,kw OR (Dysfunction, Cognitive):ab,ti,kw OR (Developmental Coordination Disorders):ab,ti,kw OR (Dysfunctions, Cognitive):ab,ti,kw OR (Cognitive Disorder):ab,ti,kw OR (Cognitive Disorders):ab,ti,kw OR (Disorder, Cognitive):ab,ti,kw OR (Dementia):ab,ti,kw OR (Dementias):ab,ti,kw |
| #6 | #4OR#5 |
| #7 | #3AND#6 |
