# Supplementary material for: Incidence of cognitive impairment post first stroke: a systematic review and meta-analysis
Source: Front Neurol. 2026 Apr 13;17:1782749. doi: 10.3389/fneur.2026.1782749 (PMC13111006; doi:10.3389/fneur.2026.1782749)
Supplement: APPENDIX 2 — Literature quality assessment. [file Supplementary_file_2.doc]

**NEWCASTLE - OTTAWA QUALITY ASSESSMENT SCALE COHORT STUDIES**

| Author | Year | **Selection** | | | | **Comparability** | **Outcome** | | | Quality score |
| --- | --- | --- | --- | --- | --- | --- | --- | --- | --- | --- |
|  |  | Representativeness of the exposed cohort | Selection of the non exposed cohort | Ascertainment of exposure | Demonstration that outcome of interest was not present at start of study | Comparability of cohorts on the basis of the design or analysis | Assessment of outcome | Was follow-up long enough for outcomes to occur | Adequacy of follow up of cohorts |  |
| Liman TG | 2011 | ★ | ★ | ★ | ★ | ★ | ★ | ★ | ★ | 8 |
| Wong GKC | 2012 | ★ | ★ | ★ | ★ | ★ | ★ |  | ★ | 7 |
| Tu QY | 2014 | ★ | ★ | ★ | ★ | ★★ | ★ | ★ |  | 8 |
| Jacquin A | 2014 | ★ | ★ | ★ | ★ | ★ | ★ |  | ★ | 7 |
| Yoon JA | 2017 | ★ | ★ | ★ | ★ | ★ | ★ | ★ |  | 7 |
| He M | 2018 | ★ | ★ | ★ | ★ | ★ | ★ | ★ |  | 7 |
| Jia H | 2020 | ★ | ★ | ★ | ★ | ★★ | ★ |  | ★ | 8 |
| Esmael A | 2021 | ★ | ★ | ★ | ★ | ★ | ★ | ★ |  | 7 |
| Xu L | 2023 | ★ | ★ | ★ | ★ | ★ | ★ |  | ★ | 7 |
| Huang YJ | 2023 | ★ | ★ | ★ | ★ | ★ | ★ | ★ |  | 7 |
| Boutros CF | 2023 | ★ | ★ | ★ | ★ | ★★ | ★ | ★ |  | 8 |
